# Are medical students in Palestine adequately trained to care for individuals with autism spectrum disorders? A multicenter cross-sectional study of their familiarity, knowledge, confidence, and willingness to learn

**DOI:** 10.1186/s12909-021-02865-8

**Published:** 2021-08-10

**Authors:** Ramzi Shawahna, Mohammad Jaber, Nourhan Yahya, Firdaous Jawadeh, Shahd Rawajbeh

**Affiliations:** 1 Department of Physiology, Pharmacology and Toxicology, Faculty of Medicine and Health Sciences, An-Najah National University, P.O. Box 7, New Campus, Building: 19, Office: 1340, Nablus, Palestine; 2 An-Najah BioSciences Unit, Centre for Poisons Control, Chemical and Biological Analyses, An-Najah National University, Nablus, Palestine; 3 Department of Medicine, Faculty of Medicine and Health Sciences, An-Najah National University, Nablus, Palestine; 4 An-Najah National University Hospital, An-Najah National University, Nablus, Palestine

**Keywords:** Autism spectrum disorders, Awareness, Knowledge, Education, Medical students

## Abstract

**Background:**

Medical students are the future workforce of physicians in primary, secondary, tertiary, and highly specialized care centers. The present study was undertaken to assess familiarity, knowledge, confidence, of medical students with regard to autism spectrum disorders (ASDs).

**Methods:**

This multicenter study was conducted in a cross-sectional design among medical students in the 3 main universities in Palestine. In addition to the sociodemographic and academic details, the questionnaire measured familiarity (8-items), knowledge (12-items), confidence and willingness to learn (5-items) with regard to ASDs.

**Results:**

The questionnaire was completed by309 medical students (response rate = 77.3 %). The median familiarity, knowledge, and confidence scores were 50 % (42.5 %, 57.5 %), 50 % (41.7 %, 66.7 %), and 60.0 % (54.0 %, 68.0 %), respectively. There was a positive moderate correlation between familiarity and knowledge scores (Spearman’s rho = 0.29, p-value < 0.001) and familiarity and confidence scores (Spearman’s rho = 0.34, p-value < 0.001). Medical students who have received a course on autism were 3.08-fold (95 % C.I. of 1.78–5.31) more likely to score ≥ 50 % on the familiarity items compared to those who did not receive a course. The medical students who were in their clinical academic stage, who received a course on ASDs, and those who interacted with individuals with ASDs were 2.36-fold (95 % C.I. of 1.34–4.18), 2.66-fold (95 % C.I. of 1.52–4.65), and 2.59-fold (95 % C.I. of 1.44–4.63) more likely to score ≥ 50 % on the knowledge items. Medical students who reported high satisfaction with their social life were 2.84-fold (95 % C.I. of 1.15-7.00) more likely to score ≥ 50 % on the confidence items.

**Conclusions:**

The present study identified considerable awareness and knowledge gaps among medical students with regard to ASDs. Medical students in this study reported low confidence in their ability to provide healthcare services to individuals with ASDs. Appropriately designed educational interventions might improve familiarity, knowledge, and confidence of medical students. More studies are still needed to investigate if such interventions can improve healthcare services for individuals with ASDs.

**Supplementary Information:**

The online version contains supplementary material available at 10.1186/s12909-021-02865-8.

## Background

Since their description by Kanner in 1943, autism spectrum disorders (ASDs) have evolved from disorders that occur rarely to one of the fastest growing developmental disorders [1, 2]. Based on data from the Centers for Disease Control and Prevention (CDC), ASDs occur at a rate of 1 in 54 children [3, 4]. Considering the high prevalence rates in every nation of the world, ASDs have now evolved as an emerging public health issue [1, 2]. Research studies have shown that ASDs are more prevalent among males compared to females [2]. Additionally, epidemiological studies have shown that ASDs were more prevalent compared to pediatric cancers, juvenile diabetes, and acquired immunodeficiency syndrome in children combined [5, 6]. Studies conducted in different countries have shown that ASDs were not linked to racial, ethnic, and/or socioeconomic groups [7]. In the absence of large epidemiological surveys, little is known on the incidence and prevalence of ASDs among the Palestinians [6, 8]. Additionally, Palestinian refugees were excluded from studies conducted in the neighboring Lebanon to estimate prevalence of ASDs which was estimated between 49 and 513 per 10,000 [9, 10]. A recent systematic review and meta-analysis estimated a pooled prevalence rate of ASDs at 0.36 % (95 % CI: 0.16–0.79 %) among Asians [11]. The study included populations from South, East, and West Asia. Awareness of ASDs among the Palestinians has been growing and the national disability survey of the Palestinian Central Bureau of Statistics included ASDs within the category of “learning difficulties” [8].

Limitations in communication, social interactions, and behavioral development are typically seen among individuals with ASDs [12, 13]. Like other neurodevelopmental disorders, ASDs are not curable. However, health regulatory authorities like the US Food and Drug Administration (FDA) have approved some pharmacological and nonpharmacological management options that aim to alleviate some of the signs and symptoms that could be associated with ASDs [14, 15]. Today, prescription of pharmacotherapeutic options to manage and alleviate behavioral symptoms of ASDs like tantrums, aggressiveness, hyperactivity, and anxiety is increasingly popular in clinical practice [14–17]. Analysis of medication prescription has shown increasing volumes of psychoactive drugs among individuals with ASDs [17, 18]. Currently, the antipsychotics risperidone and aripiprazole are approved by the FDA to alleviate behavioral symptoms of ASDs [18]. Additionally, stimulants of the central nervous system, anxiolytics, and antidepressant drugs are also used in the management of symptoms of ASDs [19, 20].

In Palestine, healthcare services for individuals with ASDs are provided by 4 main sectors: (1) the public sector (healthcare facilities of the Palestinian Ministry of Health), (2) the private sector (for-profit healthcare facilities), (3) non-governmental organizations (NGOs), and (4) healthcare facilities of the United Nations Relief and Works Agency (UNRWA) [6, 8]. As the public healthcare services often fall short for some diagnostic and health needs of the patients, therefore, diagnostic, psychosocial support, and rehabilitation services to individuals with ASDs are predominantly provided by the private sector and NGOs. The Ministry of Health often subsidize diagnostic and care services within the private sector or NGOs to the needy patients who cannot afford the costs in case these services were not provided by the public healthcare facilities.

Although in contemporary practice, individuals with ASDs might receive services from specialty providers like behavioral optometrists, clinical psychologists, counsellors/psychotherapists, psychiatrists, and occupational therapists, physicians are the backbone of healthcare delivery in the majority of the healthcare systems around the world. In resource deficient settings and in the absence of multi-healthcare provider approaches to healthcare, physicians are supposed to make diagnosis, prescribe medications, and guide the treatment plans for patients including those with ASDs. Diagnosis and management of individuals with ASDs can be highly challenging in clinical practice [21, 22]. This is particularly true for pharmacotherapy as individuals with ASDs were more susceptible to the side effects of these pharmacotherapeutic modalities compared to individuals without ASDs [23, 24]. Additionally, individuals with ASDs were shown to exhibit paradoxical reactions to some drugs [25]. Medical students are the future workforce of physicians in all specialties. With the increasing prevalence of ASDs, future physicians are expected to encounter and provide care for this segment of the society. Therefore, future physicians should be knowledgeable of ASDs [26]. Inadequately trained physicians or physicians with inadequate familiarity and knowledge of ASDs are less likely to provide quality care for individuals with ASDs compared with familiar and knowledgeable physicians. Provision of suboptimal care might have serious consequences on the health and quality of life of the affected patients. Unfortunately, studies have shown that ASDs are still not adequately understood by healthcare providers including physicians who are supposed to be knowledgeable of health issues of people with ASDs [6, 27]. Physicians are supposed to make or help make diagnosis of ASDs, provide counseling to families or caregivers of the affected individuals, manage symptoms, and make appropriate referrals to specialty services when needed. With the increasing prevalence of ASDs, those individuals are increasingly encountered in primary healthcare practice. Physicians should also be able to help individuals and their families to benefit from resources allocated within the community for the individuals and their families.

Assessing familiarity, knowledge, confidence, and willingness to learn of medical students on the different aspects of ASDs can be important in measuring and benchmarking quality of medical training and preparing future physicians who would provide healthcare to individuals with ASDs. Familiarity, knowledge, confidence, and willingness to learn of medical students on ASDs were not previously assessed among medical students trained in Palestine. Therefore, this study was conducted to: (a) assess familiarity of the medical students with individuals with ASDs, (b) assess familiarity of the medical students with symptoms, diagnosis, treatment options, and community resources to help people with ASDs and their families, (c) assess knowledge of the medical students with etiology, prevalence, and treatment of ASDs, (d) assess confidence of the medical students to provide care/counsel people with ASDs and willingness to learn about ASDs, (e) investigate correlations between familiarity, knowledge, and confidence scores of the medical students, (f) investigate associations between sociodemographic and academic variables of the students with familiarity, knowledge, and confidence scores, and (g) identify predictors of higher familiarity, knowledge, and confidence scores.

## Methods

### Study context and settings

This multicenter study was conducted among Palestinian medical students in the three main universities in Palestine. The study was conducted in the context of assessing training of future physicians to care for individuals with ASDs. The Doctor of Medicine (MD) program offered at Palestinian universities consists of 130 credit hours of basic courses and 135 credit hours of hospital/clinic-based training. The students often complete the MD program in 6 academic years. Fresh graduates have to receive a 1-year internship before they could be licensed to practice medicine in Palestine.

### Study design

This multicenter study was conducted in a cross-sectional design using a questionnaire as the study tool. Therefore, the study was conducted and reported in adherence to the guidelines for reporting cross-sectional studies in which a questionnaire was used as the study tool [28–30]. Adherence to the guidelines is shown in Supplementary Table S1. The guidelines ensured reporting the study design, objectives, settings, recruitment process, sampling, participants, representativeness, study tool, reliability/validity, psychometrics, data analysis, main results, interpretation of the findings, discussion of the strengths, limitations, and generalizability.

### The study population, sample size, and inclusion criteria

This study population was medical students in the 3 major universities in Palestine. The number of medical students needed in this study was estimated using a sample size estimator that is accessible online (www.raosoft.com). The sample size was estimated for a population of 3,500 medical students. The number of medical students needed for this study was estimated at a 95 % confidence interval (C.I.) with a 5 % margin of error. The sample size needed was 347 students.

Medical students were eligible for inclusion in this study if they were: (a) 18 years and older, (b) enrolled in an MD program in a Palestinian university, (c) willing to respond to items in a questionnaire, and (d) willing to provide informed consent. The medical students were approached and invited by the field researchers from the 3 major universities in Palestine. The field researchers explained the study objectives to the potential participants and provided them with the questionnaire. The medical students consented to participate without any financial or academic incentives.

### The study questionnaire

The questionnaire used in this study was adopted and modified from previous studies that were conducted to assess awareness and knowledge of healthcare providers with regard to people with ASDs [5, 6, 31, 32]. The questionnaire used in this study consisted of 4 sections. Section 1 collected the sociodemographic and academic variables of the medical students like: gender, place of residence, and academic years/stage. The medical students were also asked to state if they have had a course/part of a course on ASDs, or had interacted with somebody with ASDs, and who was that person. Additionally, the medical students were asked to state if they were satisfied with their academic achievement, satisfied with their financial status, and satisfied with their social status. Section 2 contained 8 items to collect familiarity of the medical students with symptoms, diagnosis, treatment options, and community resources to help people with ASDs and their families. On each item, the medical students had to rate their familiarity on a Likert-scale of 1–5 (1 = completely unfamiliar, 5 = completely familiar). Section 3 contained 12 items to measured knowledge of medical students with regard to etiology, prevalence, and treatment of ASDs. On each knowledge item, the medical students had to respond by either true/false/I don’t know. Section 4 contained 5 items to measure confidence of the medical students to confidence of the medical students to provide care/counsel people with ASDs and willingness to learn about ASDs. On each item, the medical students had to indicate their level of disagreement/agreement on a Likert-scale of 1–5 (1 = strongly disagree, 5 = strongly agree).

### Pilot testing of the questionnaire

Before the questionnaire was used in the larger study, the questionnaire was pilot tested with 18 medical students who did not take part in the larger study. The medical students were asked to read and rated the items for clarity and comprehension. Some items were rephrased for clarity based on the feedback obtained in the pilot testing. The 18 medical students were asked to respond to the questionnaire in two consecutive rounds. The time between both administrations was 30 min – 2 h. Stability of scores was assessed using the test-retest method. The internal consistency (item relatedness) was assessed using Cronbach’s alpha. A Pearson’s correlation coefficient (*r*) of > 0.8 indicated acceptable reliability and a Cronbach’s alpha of > 60 % indicated acceptable internal consistency. The internal consistency of familiarity, knowledge, and confidence items were assessed separately.

### Data analysis

Familiarity scores were summed and could range from 8 to 40. For each correct answer, students were awarded 1 point and 0 point for each incorrect/I don’t know answer. Knowledge scores were summed and could range from 0 to 12. Confidence scores were summed and could range from 5 to 25. All scores were also converted into percentages.

The difficulty index (D) was used to psychometrically assess the difficulty of each knowledge item. The D values were ratios between correct answers/total number of answers for each item. The knowledge items were attributed as follows: (a) a very difficult class: when 20 % < D, (b) difficult class: when 20 % ≤ D < 40 %, (c) moderate difficulty class: when 40 % ≤ D < 60 %, (d) easy class: when 60 % ≤ D < 80 %, and (e) very easy class: when D ≥ 80 % [6].

The data were assessed for normal distribution using the Kolmogorov–Smirnov test. As the data were not normally distributed, the data were expressed using medians and interquartile range (Q1, Q3). Spearman’s rank correlations, Pearson’s Chi-Square test, and/or Fisher’s exact test were used to compare categorical data, as appropriate. To control potentially confounding variables and assess the strength of association, a multiple logistic regression model was used to determine predictors of scoring ≥ 50 % on the familiarity, knowledge, and confidence items. All variables were retained in the multiple logistic regression model. A *p*-value < 0.05 was considered as statistically significant.

### Ethics approval and consent to participate

This multicenter cross-sectional study was conducted in adherence to the international guidelines and regulations including those in the Declaration of Helsinki and the ones followed at An-Najah National University. The study received ethical approval from the Institutional Review Board of An-Najah National University. The medical students who participated in this study provided written informed consent.

## Results

### Reliability and internal consistency of the items in the questionnaire

The Pearson’s *r* of the scores in both rounds was 0.97 with a *p*-value < 0.001 which indicated excellent reliability (stability of scores over a short period of time). The Cronbach’s alpha values were 0.81, 0.68, and 0.66 for the familiarity, knowledge, and confidence items, respectively. The values indicated good relatedness (internal consistency) of the items used for each dimension.

### Sociodemographic and academic variables of the medical students

Of the 400 medical students invited, the questionnaire was completed by 309 medical students, giving a response rate of 77.3 %. The sociodemographic and academic variables of the medical students who participated in this study are shown in Table 1. Of the study participants, female students were well represented (71.8 %) which reflected the high percentage of female medical students in the MD program in the Palestinian universities, 58.6 % of the medical students lived in urban areas, 61.2 % were in their clinical stage of the MD program (4th to 6th year), the vast majority (97.7 %) of the medical students were satisfied with their academic achievement, 94.2 % were satisfied with their financial status, 88.7 % were satisfied with their social status, 65.7 % reported that they had a course/part of a course on ASDs, and 34.0 % reported that they had interacted with someone with ASDs (Table 1).

**Table 1:** Sociodemographic and academic variables of the medical students who participated in this study (*n = 309*)

Variable	n	%
**Gender**		
Male	87	28.2
Female	222	71.8
**Place of residence**		
Countryside	128	41.4
Urban	181	58.6
**Academic stage**		
Basic stage (1st to 3rd year)	120	38.8
Clinical stage (4th to 6th year)	189	61.2
**Self-rated satisfaction with academic achievement**		
Not satisfied	7	2.3
Satisfied	302	97.7
**Self-rated satisfaction with financial status**		
Not satisfied	18	5.8
Satisfied	291	94.2
**Self-rated satisfaction with social status**		
Not satisfied	35	11.3
Satisfied	274	88.7
**Had a course/part of a course on ASDs**		
No	106	34.3
Yes	203	65.7
**Interacted with somebody with ASDs**		
No	204	66.0
Yes	105	34.0

### Interaction with somebody with ASDs

Of the medical students who reported interacting with someone with ASDs, 40.0 % reported interacting with a relative with ASDs, 38.1 % reported interacting with somebody from the neighborhood with ASDs, and 23.8 % interacted with an individual with ASDs during their training. Details of the responses of the medical students are shown in Table 2.

**Table 2:** Interaction with somebody with ASDs

Who was the person with autism spectrum disorder	n*	%*
A relative	42	40.0
Somebody from the neighborhood	40	38.1
An individual during my training	25	23.8
A family member	16	15.2
A relative of a friend	14	13.3

^*^Percentages were calculated from the number of the medical students who reported interacting with somebody with ASDs (*n* = 105). The numbers do not sum to the total number of the medical students who participated in this study and the percentages do not sum to 100 %

### Familiarity of the medical students with symptoms, diagnosis, treatment options, and community resources to help people with ASDs and their families

There was a positive moderate correlation between familiarity scores and knowledge scores (Spearman’s rho = 0.29, *p*-value < 0.001). Similarly, there was a moderate positive correlation between familiarity scores and confidence scores (Spearman’s rho = 0.34, *p*-value < 0.001).

The median familiarity score was 50% with an interquartile range of (42.5%, 57.5%). The distribution of the responses of the medical students on the familiarity items is shown in Table 3. Worrisomely, a considerable percentage of the medical students reported inadequate familiarity with symptoms, diagnosis, treatment options, and community resources to help people with ASDs and their families as indicated by the percentage of the medical students who rated their familiarity on the Likert-scale 1-3 (Not familiar at all to somewhat familiar) and the low percentage of students who rated their familiarity on the Likert-scale 4-5 (familiar to completely familiar). Of the medical students, 78.6% reported in adequate familiarity with the symptoms of ASDs, 92.2% reported inadequate familiarity with the tools used to diagnose ASDs, 81.9% reported inadequate familiarity with the classes of medications that can be used in treating the symptoms of ASDs, 72.5% reported inadequate familiarity with the behaviors associated with ASDs that medications seek to alleviate, 92.2% reported inadequate familiarity with the doses of medications used in the treatment of ASDs symptoms, 87.4% reported inadequate familiarity with the various side effects produced by medications used in the treatment of ASDs symptoms, 84.8% reported inadequate familiarity with how to help families sort through information to make informed decisions about their child with ASDs, and 87.1% reported inadequate familiarity with community resources in their region that can be used for referral of a child who is exhibiting symptoms commonly associated with ASDs.

**Table 3:** Familiarity of the medical students with symptoms, diagnosis, treatment options, and community resources to help people with ASDs and their families

		Not familiar at all		Not familiar		Somewhat familiar		Familiar		Completely familiar	
**#**	**Familiarity item**	**n**	**%**	**n**	**%**	**n**	**%**	**n**	**%**	**n**	**%**
	**How would you rate your familiarity with**										
1	Different symptoms of ASDs	13	4.2	63	20.4	167	54.0	53	17.2	13	4.2
2	Different tools used to diagnose ASDs	50	16.2	142	46.0	93	30.1	18	5.8	6	1.9
3	Different classes of drugs (e.g., antidepressants, antipsychotics, central nervous system stimulants) that can be used in the management of the symptoms of ASDs	38	12.3	107	34.6	108	35.0	52	16.8	4	1.3
4	Specific behaviors associated with ASDs that drugs seek to alleviate (e.g., self-injury, hyperactivity, and obsessive-compulsive disorder)	24	7.8	68	22.0	132	42.7	68	22.0	17	5.5
5	Doses of drugs used in the management of symptoms of ASDs	95	30.7	143	46.3	47	15.2	20	6.5	4	1.3
6	Various side effects produced by drugs used in the management of symptoms of ASDs (e.g., irritation, sedation, and extrapyramidal symptoms)	47	15.2	131	42.4	92	29.8	34	11.0	5	1.6
7	How to help families sort through information to make informed decisions about their child with ASDs	30	9.7	121	39.2	111	35.9	42	13.6	5	1.6
8	Community resources available in the region that can be used for referral of a child who is exhibiting symptoms commonly associated with ASDs	44	14.2	125	40.5	100	32.4	33	10.7	7	2.3

### Knowledge of the medical students with regard to etiology, prevalence, and treatment of ASDs

On the knowledge items, the median score was 50 % with an interquartile range of (41.7 %, 66.7 %). Detailed responses of the medical students on each knowledge item are shown in Table 4.

**Table 4:** Knowledge of the medical students with etiology, prevalence, and treatment of ASDs

		True		False		I don’t know		
**#**	**Knowledge item**	**n**	**%**	**n**	**%**	**n**	**%**	**D**
1	ASDs are developmental disorders?	**187**	**60.5**	57	18.4	65	21.0	Easy
2	Children with ASDs have impairments in social interaction, communication or language, and behavioral development?	**288**	**93.2**	6	1.9	15	4.9	Very easy
3	ASDs occur more commonly among males than females?	**123**	**39.8**	27	8.7	159	51.5	Difficult
4	ASDs are more prevalent than juvenile diabetes?	**40**	**12.9**	58	18.8	211	68.3	Very difficult
5	ASDs are more prevalent than Down syndrome?	**66**	**21.4**	82	26.5	161	52.1	Difficult
6	ASDs are curable?	45	14.6	**193**	**62.5**	71	23.0	Easy
7	Risperidone and aripiprazole have been approved by the health authorities for the treatment of irritability associated with ASDs?	**53**	**17.2**	13	4.2	243	78.6	Very difficult
8	Vaccines can cause ASDs?	23	7.4	**207**	**67.0**	79	25.6	Easy
9	ASDs exist only in childhood?	59	19.1	**199**	**64.4**	51	16.5	Easy
10	ASDs are caused because of emotionally distant, rejecting parents?	102	33.0	**133**	**43.0**	74	23.9	Moderate
11	Genetic factors play a major role in the etiology of ASDs?	**219**	**70.9**	31	10.0	59	19.1	Easy
12	ASDs are rare disorder?	37	12.0	**199**	**64.4**	73	23.6	Easy

Correct answers are boldface

Despite some high awareness areas like awareness of impairments in social interaction, communication or language, and behavioral development among children with ASDs (item #2) which was correctly answered by 93.2 % of the medical students. Several knowledge gaps were identified as indicated by the number of incorrect answers/I don’t know answers. Of the medical students, 70.9 % could correctly answer the item on the role of genes in ASDs (item # 11), 67.0 % could correctly answer the item on the myth that vaccines can cause autism (item #8), 64.4 % could correctly answer the item on the existence of ASDs beyond childhood (item # 9), 64.4 % could correctly answer the item on the high prevalence of ASDs (item # 12), 62.5 % could correctly answer the item on the incurability of ASDs (item # 6), 60.5 % could correctly answer the item on the spectrum disorders as developmental disorders (item #1), 43.0 % could correctly answer the item on the myth that ASDs are caused by emotionally distant, rejecting parents (item # 10), 39.8 % could correctly answer the item on the higher incidence of ASDs among males (item # 3), 21.4 % could correctly answer the item on the higher prevalence of ASDs compared to Down syndrome (item # 5), 17.2 % could correctly answer the item on the use of risperidone and aripiprazole in the treatment of irritability associated with ASDs (item #7), and 12.9 % could correctly answer the item on the higher prevalence of ASDs compared to juvenile diabetes (item # 4).

### Confidence of the medical students to provide care/counsel people with ASDs and willingness to learn about ASDs

The median confidence score was 60.0 % with an interquartile range of (54.0 %, 68.0 %). Detailed responses of the medical students on each confidence and willingness to learn item are shown in Table 5. 81.9 % of the medical students reported not feeling adequately confident in their ability to counsel parents about the diagnosis of their child with ASDs, 90.6 % of the medical students reported not feeling adequately confident in their ability to prescribe medications for their child with ASDs, and 79.6 % of the medical students reported not feeling adequately confident in their ability to counsel parents about the medication profile and side effects of prescriptions being used for the treatment of their child with ASDs. On the other hand, 74.8 % of the medical students reported that they might benefit from taking additional education or training in the area of ASDs and 81.9 % of the medical students reported that the medical school curriculum should include a separate course or lecture in the area of ASDs.

**Table 5:** Confidence of the medical students to provide care/counsel people with ASDs and willingness to learn about ASDs

		Strongly disagree		Disagree		Neutral		Agree		Strongly agree	
**#**	**Confidence item**	**n**	**%**	**n**	**%**	**n**	**%**	**n**	**%**	**n**	**%**
1	I feel confident in my abilities to counsel parents with regard to diagnosis of their child with ASDs	32	10.4	82	26.5	139	45.0	48	15.5	8	2.6
2	I feel confident in my abilities to prescribe drugs for their child with ASDs	101	32.7	97	31.4	82	26.5	22	7.1	7	2.3
3	I feel confident in my abilities to counsel parents about the drug profile and side effects of prescriptions being used for the treatment of their child with ASDs	54	17.5	101	32.7	91	29.4	49	15.9	14	4.5
4	I feel that I could benefit from taking additional education or training in the area of ASDs	8	2.6	21	6.8	49	15.9	136	44.0	95	30.7
5	I feel that the medical school curriculum should include a separate course or lecture in the area of ASDs	7	2.3	7	2.3	42	13.6	127	41.1	126	40.8

### Associations between sociodemographic and academic variables of the students with familiarity, knowledge, and confidence scores

Associations between sociodemographic and academic variables of the students with familiarity, knowledge, and confidence scores are shown in Table 6.

**Table 6:** Association between sociodemographic and academic variables of the students with familiarity, knowledge, and confidence scores

	Familiarity						Knowledge						Confidence					
	< 50 %		≥ 50 %				< 50 %		≥ 50 %				< 50 %		≥ 50 %			
Variable	n	%	n	%	Chi/Fisher	***p***-value	n	%	n	%	Chi/Fisher	***p***-value	n	%	n	%	Chi/Fisher	***p***-value
**Gender**																		
Male	38	12.3	49	15.9	0.00	1.000	30	9.7	57	18.4	0.04	0.894	12	3.9	75	24.3	0.08	0.851
Female	97	31.4	125	40.5			74	23.9	148	47.9			28	9.1	194	62.8		
**Place of residence**																		
Countryside	53	17.2	75	24.3	0.46	0.561	41	13.3	87	28.2	0.26	0.627	20	6.5	108	35.0	1.39	0.302
Urban	82	26.5	99	32.0			63	20.4	118	38.2			20	6.5	161	52.1		
**Academic stage**																		
Basic stage (1st to 3rd year)	59	19.1	61	19.7	2.39	0.128	58	18.8	62	20.1	18.93	< 0.001	13	4.2	107	34.6	0.78	0.393
Clinical stage (4th to 6th year)	76	24.6	113	36.6			46	14.9	143	46.3			27	8.7	162	52.4		
**Satisfaction with academic achievements**																		
Not satisfied	4	1.3	3	1.0	0.53	0.703	3	1.0	4	1.3	0.27	0.691	3	1.0	4	1.3	5.67	0.049
Satisfied	131	42.4	171	55.3			101	32.7	201	65.0			37	12.0	265	85.8		
**Satisfaction with financial status**																		
Not satisfied	10	3.2	8	2.6	1.09	0.334	8	2.6	10	3.2	1.00	0.441	3	1.0	15	4.9	0.23	0.419
Satisfied	125	40.5	166	53.7			96	31.1	195	63.1			37	12.0	254	82.2		
**Satisfaction with social status**																		
Not satisfied	17	5.5	18	5.8	0.38	0.589	14	4.5	21	6.8	0.71	0.448	9	2.9	26	8.4	5.69	0.029
Satisfied	118	38.2	156	50.5			90	29.1	184	59.5			31	10.0	243	78.6		
**Had a course/part of a course on ASDs**																		
No	65	21.0	41	13.3	20.39	< 0.001	56	18.1	50	16.2	26.56	< 0.001	17	5.5	89	28.8	1.37	0.284
Yes	70	22.7	133	43.0			48	15.5	155	50.2			23	7.4	180	58.3		
**Interacted with people with ASDs**																		
No	96	31.1	108	35.0	2.77	0.115	79	25.6	125	40.5	6.91	0.011	26	8.4	178	57.6	0.02	1.000
Yes	39	12.6	66	21.4			25	8.1	80	25.9			14	4.5	91	29.4		

Chi/Fisher: Pearson’s Chi-square/Fisher’s Exact test

Scoring ≥ 50% on the familiarity items was significantly associated with having a course/part of a course on ASDs (Pearson Chi-Square/Fisher's Exact test = 20.39, *p*-value < 0.001). Other sociodemographic and academic variables were not significantly associated with the familiarity scores. Scoring ≥ 50% on the knowledge items was significantly associated with academic stage (Pearson Chi-Square/Fisher's Exact test = 18.93, *p*-value < 0.001), having a course/part of a course on ASDs (Pearson Chi-Square/Fisher's Exact test = 26.56, *p*-value < 0.001), and having interacted with someone with autism spectrum disorder (Pearson Chi-Square/Fisher's Exact test = 6.91, *p*-value = 0.011). Other sociodemographic and academic variables were not significantly associated with the knowledge scores. Scoring ≥ 50% on the confidence and willingness items was significantly associated with satisfaction with academic achievement (Pearson Chi-Square/Fisher's Exact test = 5.67, *p*-value = 0.049) and satisfaction with social status (Pearson Chi-Square/Fisher's Exact test = 5.69, *p*-value = 0.029). Other sociodemographic and academic variables were not significantly associated with the confidence and willingness scores.

Logistic regression showed that the medical students who have had a course/part of a course on ASDs were 3.08-fold (95 % C.I. of 1.78–5.31) more likely to score ≥ 50 % on the familiarity items compared to those who did not have a course/part of a course on ASDs. The medical students who were in their clinical academic stage (4th to 6th year) were 2.36-fold (95 % C.I. of 1.34–4.18) more likely to score ≥ 50 % on the knowledge items compared to those who were in their basic stage (1st to 3rd year), those who have had a course/part of a course on ASDs were 2.66-fold (95 % C.I. of 1.52–4.65) more likely to score ≥ 50 % on the knowledge items compared to those who did not have a course/part of a course on ASDs, and the medical students who had interacted with someone with ASDs were 2.59-fold (95 % C.I. of 1.44–4.63) more likely to score ≥ 50 % on the knowledge items compared to those who had not interacted with someone with ASDs. Medical students who reported high satisfaction with their social life were 2.84-fold (95 % C.I. of 1.15-7.00) more likely to score ≥ 50 % on the confidence items compared to those who reported low satisfaction with their social life. Details of the logistic regression model are shown in Table 7.

**Table 7:** Factors associated with familiarity, knowledge, and confidence scores

							95 % C.I. for O.R.	
Dimension	Variable	β	S.E.	Wald	***p***-value	O.R.	Lower	Upper
**Familiarity**	Gender	0.02	0.27	0.01	0.941	1.02	0.60	1.73
	Place of residence	0.22	0.25	0.77	0.381	1.24	0.77	2.01
	Academic stage	-0.04	0.28	0.03	0.874	0.96	0.56	1.65
	Satisfaction with academic achievements	-0.32	0.83	0.15	0.702	0.73	0.14	3.68
	Satisfaction with financial status	-0.48	0.53	0.82	0.366	0.62	0.22	1.75
	Satisfaction with social status	0.18	0.38	0.21	0.646	1.19	0.56	2.51
	Had a course/part of a course on ASDs	1.12	0.28	16.31	< 0.001	3.08	1.78	5.31
	Interacted with people with ASDs	0.47	0.26	3.28	0.070	1.60	0.96	2.68
**Knowledge**	Gender	-0.06	0.29	0.04	0.851	0.95	0.53	1.68
	Place of residence	0.28	0.27	1.07	0.301	1.32	0.78	2.24
	Academic stage	0.86	0.29	8.77	0.003	2.36	1.34	4.18
	Satisfaction with academic achievements	0.00	0.84	0.00	0.998	1.00	0.19	5.16
	Satisfaction with financial status	-0.42	0.56	0.57	0.451	0.65	0.22	1.97
	Satisfaction with social status	0.30	0.40	0.56	0.456	1.35	0.61	2.98
	Had a course/part of a course on ASDs	0.98	0.29	11.68	0.001	2.66	1.52	4.65
	Interacted with people with ASDs	0.95	0.30	10.20	0.001	2.59	1.44	4.63
**Confidence**	Gender	-0.31	0.39	0.62	0.432	0.73	0.34	1.58
	Place of residence	-0.46	0.36	1.66	0.198	0.63	0.31	1.27
	Academic stage	-0.76	0.42	3.24	0.072	0.47	0.21	1.07
	Satisfaction with academic achievements	-1.58	0.87	3.29	0.070	0.21	0.04	1.14
	Satisfaction with financial status	-0.07	0.71	0.01	0.918	0.93	0.23	3.71
	Satisfaction with social status	1.04	0.46	5.13	0.023	2.84	1.15	7.00
	Had a course/part of a course on ASDs	0.65	0.40	2.66	0.103	1.91	0.88	4.15
	Interacted with people with ASDs	-0.17	0.37	0.21	0.647	0.84	0.40	1.75

*C.I.* Confidence interval, *O.R.* Odds ratio, *S.E.* Standard error

## Discussion

Medical students are the future workforce of physicians in all specialties in primary, secondary, tertiary, and highly specialized care centers. As a backbone of healthcare provision in any healthcare system, physicians should be adequately trained to care for individuals including those with ASDs. This multicenter study is the first attempt to assess familiarity, knowledge, confidence, and willingness to learn of medical students on the different aspects of ASDs. Several awareness and knowledge gaps were identified among the medical students trained in Palestinian universities with regard to symptoms, diagnosis, treatment options, and community resources to help people with ASDs and their families.

Findings of this study showed that familiarity of medical students with regard to symptoms, diagnosis, treatment options, and community resources to help people with ASDs and their families was inadequate as indicated by the median familiarity score. In this study, the median familiarity score was 50 % (42.5 %, 57.5 %). Findings of this study were consistent with those reported among other healthcare providers like practicing pharmacists in Palestine and nurses in Virginia [6, 33]. On the other hand, familiarity of medical students in this study were relatively lower compared to pharmacists and pharmacy students in Mississippi as indicated by the percentages of participants rating familiarity items as not familiar at all or not familiar compared to the percentages of those who rated the familiarity items as familiar or completely familiar [5, 34]. In this study, a worrisomely high percentage of medical students (78.6 %) reported inadequate familiarity with signs and symptoms of ASDs. Additionally, the vast majority of the medical students in this study reported inadequate familiarity with the diagnostic tools used in the diagnosis of ASDs. In clinical practice, signs and symptoms are important for differential diagnosis of diseases and disorders [35, 36]. Physicians and future physicians should be able to recognize signs and symptoms of diseases and disorders and should be able to make diagnosis. Inability to recognize signs and symptoms of a disease or disorder would hinder physicians from making correct diagnosis and subsequently manage the disease. Today, medications like risperidone and aripiprazole are approved for the management of some behavioral symptoms of ASDs [14–17]. While data from real-world practice showed that aripiprazole was generally effective and well-tolerated in the management of irritability associated with ASDs among children and adolescents [37], other studies found that relapse rates were not different between pediatric patients with ASDs who were randomized to receive aripiprazole compared to those who were randomized to receive placebo [38]. These findings suggested re-evaluating the use of aripiprazole following a period of stabilizing the patients. In this study, the majority of the medical students reported inadequate familiarity with the medications used in the treatment of behavioral symptoms of ASDs, their doses, and adverse effects. This might have future implications on the quality of healthcare provided to individuals with ASDs as physicians are the ones who prescribe medications to patients. In many healthcare systems, physicians are the only authorized healthcare providers to prescribe medications to patients [39]. Physicians are supposed to prescribe the right medications, with right doses, at the right frequency, and for the right period of time. Physicians should also be knowledgeable of the potential adverse effects that could be associated with the use of these medications [40]. Physicians are the primary source of information for patients in many healthcare systems around the world [41]. Therefore, physicians should be able to help individuals with ASDs and their families/caregivers sort through information and make informed decisions. In this study, the majority of medical students reported inadequate familiarity with community resources in their region and how to help families make informed decisions. This might affect their ability to perform their expected roles in caring for individuals with ASDs.

When knowledge of medical students with regard to etiology, prevalence, and treatment of ASDs was tested, several low awareness areas were identified. In this study, the majority of the medical students either answered incorrectly or did not know that ASDs were more prevalent than juvenile diabetes (87.1 %) and Down syndrome (78.6 %). Surprisingly, these percentages were higher than those reported among community pharmacists in Palestine, pharmacists, and pharmacy students in Mississippi [5, 6, 34]. In this study, the majority of medical students (82.8 %) either answered incorrectly or did not know that risperidone and aripiprazole were approved for the treatment of the behavioral symptoms of ASDs. Despite some high awareness areas like awareness of impairments in social interaction, communication or language, and behavioral development among children with ASDs which was correctly answered by 93.2 % of medical students. Several knowledge gaps were identified as indicated by the number of incorrect answers/I don’t know answers. Together, results reported in this study highlighted the need to improve knowledge of future physicians with regard to etiology, prevalence, and treatment of autism spectrum. In this study, 33 % of medical students did not correctly answer the question on the myth that linked vaccines to ASDs. Physicians are highly trusted by the society. Therefore, physicians are in key position to influence decisions of parents to vaccinate their children [42]. In different countries around the world, myths have always augmented vaccine hesitancy [43]. Findings of this study highlighted the need to dispel this myth among medical students.

In this study, receiving a course/part of a course on ASDs was a predictor of higher familiarity and knowledge with regard to ASDs. Findings of this study were consistent with those previously reported among practicing pharmacists in Palestine [6]. Previous studies have shown that receiving courses can increase familiarity and knowledge of healthcare providers including physicians on different healthcare conditions [27, 44, 45]. Interestingly, medical students who interacted with people with ASDs were more likely to score 50 % and more in the knowledge test. These findings were not surprising as interacting with patients with a certain condition might stimulate curiosities and knowledge seeking behavior [46, 47]. In this study, medical students who reported higher satisfaction with their social life were more likely to score 50 % and more on the confidence and willingness to learn items. Probably, those students were more social and willing to interact with people including those with ASDs.

### Strengths and limitations

Results of this study should be interpreted considering a number of strength and limitation points. First, this is the first multicenter study to assess familiarity, knowledge, confidence, and willingness to learn of medical students about symptoms, diagnosis, treatment options, and community resources to help people with ASDs and their families. Second, medical students who participated in this study were diversified in terms of gender, academic year, place of residence, and interaction with people with ASDs. Third, the study tool used in this study was previously validated and used among healthcare providers in different settings. Although the tool was previously assessed for reliability and internal consistency, the tool was revalidated among medical students using appropriate statistical tests. Fourth, data obtained in this study were analyzed consecutively using multiple statistical tests. On the other hand, the study had a number of limitations. First, the questionnaire used in this study oversimplified the reality of familiarity and knowledge of ASDs. More studies are still needed to understand what factors contribute to lack of knowledge of ASDs among the medical students. It is noteworthy mentioning that behavioral, communicational, and rehabilitation services are the primary services provided to individuals with ASDs. These services are often provided by specialty providers. Second, the questionnaire used multiple-choice questions. Multiple-choice questions are biased by design as they provide pre-conceived answers that the participant would choose from. Probably, an objective structured clinical examination could provide more insights into the ability of medical students to assess individuals with ASDs. Third, the sample size of this study was relatively small. However, medical students were recruited from the 3 major universities in Palestine. Additionally, the sample size used in this study was larger than those used in previous studies that assessed knowledge of healthcare providers with regard to ASDs [5, 34].

Findings of this study could be informative to decision and policy makers in academia, healthcare, advocacy groups, and professional associations who might need to design appropriate measures to improve familiarity, knowledge, and confidence of medical students in caring for individuals with ASDs.

## Conclusion

Medical students are the future workforce of physicians in all healthcare settings including those providing healthcare services to individuals with ASDs. The present study identified considerable awareness and knowledge gaps among medical students with regard to ASDs. Medical students in this study reported low confidence in their ability to provide healthcare services for children with ASDs. Appropriately designed educational interventions might improve familiarity, knowledge, and confidence of medical students. More studies are still needed to investigate if such interventions can improve healthcare services for individuals with ASDs

## Supplementary Information

**Additional file 1.**

## Data Availability

All data relevant to this study were included in the manuscript or provided as supplementary materials. The datasets used and/or analyzed during the current study are available from the corresponding author on reasonable request.
