# Supplementary material for: Are medical students in Palestine adequately trained to care for individuals with autism spectrum disorders? A multicenter cross-sectional study of their familiarity, knowledge, confidence, and willingness to learn
Source: BMC Med Educ. 2021 Aug 10;21:424. doi: 10.1186/s12909-021-02865-8 (PMC8356397; doi:10.1186/s12909-021-02865-8)
Supplement: Supplementary file 1 — Additional file 1. [file 12909_2021_2865_MOESM1_ESM.docx]

**Supplementary materials for the manuscript**

**Are medical students in Palestine adequately trained to care for patients with autism spectrum disorders? A multicenter cross-sectional study of their familiarity, knowledge, confidence, and willingness to learn**

Ramzi Shawahna^1,2*^, Mohammad Jaber^3,4^, Nourhan Yahya^3^, Firdaous Jawadeh^3^, Shahd Rawajbeh^3^

^1^Department of Physiology, Pharmacology and Toxicology, Faculty of Medicine and Health Sciences, An-Najah National University, Nablus, Palestine

^2^An-Najah BioSciences Unit, Centre for Poisons Control, Chemical and Biological Analyses, An-Najah National University, Nablus, Palestine

^3^Department of Medicine, Faculty of Medicine and Health Sciences, An-Najah National University, Nablus, Palestine

^4^An-Najah National University Hospital, An-Najah National University, Nablus, Palestine

**^*^Correspondence:**

Ramzi Shawahna, PhD, Department of Physiology, Pharmacology and Toxicology, Faculty of Medicine & Health Sciences, New Campus, Building: 19, Office: 1340, An-Najah National University, P.O. Box 7, Nablus, Palestine

Phone: + (970) 923 45113 ext 2772

Phone: + (970) 92349739

Email: [ramzi_shawahna@hotmail.com](mailto:ramzi_shawahna@hotmail.com)

**Supplementary Table S1:** Adherence to the guidelines of reporting of cross-sectional studies in which a questionnaire was used as the study tool [[1-3](#_ENREF_1)]

| **Checklist Item** | **Place in the manuscript** |
| --- | --- |
| **Title and abstract** |  |
| Design of the study stated | The title and abstract indicate the study design. Please see the title page and the abstract. |
|  | Please see the abstract. The abstract was formatted in compliance with the journal’s style |
| **Introduction** |  |
| Background provided | Provided in the Background section |
| Purpose/aim of paper explicitly stated | Provided in the last paragraph of the Background section |
| **Methods** |  |
| Study design | Methods section: under Study design |
| Setting | Methods section: under Study context and settings |
| **Recruitment process and sample description** |  |
| Participants | Methods section: under the study population, sample size, and inclusion criteria |
| Description of the population and sample frame | Methods section: under the study population, sample size, and inclusion criteria |
| Description of representativeness of the sample | Methods section: under the study population, sample size, and inclusion criteria |
| Sample size calculation or rationale/justification presented | Methods section: under the study population, sample size, and inclusion criteria |
| Incentives | Methods section: under the study population, sample size, and inclusion criteria |
| **Tool of measurement** |  |
| Description of the questionnaire | Methods section: under the study questionnaire |
| References to original work provided | Methods section: under the study questionnaire |
| Reliability and validity reported | Methods section: under Pilot testing of the questionnaire |
| Psychometric properties presented | Methods section: under Data analysis |
| Description of the scoring procedures provided | Methods section: under Data analysis |
| **Survey Administration** |  |
| Description of who approached potential participants | Methods section: under the study population, sample size, and inclusion criteria |
| **Analysis** |  |
| Methods of data analysis | Methods section: under Data analysis |
| **Statistical analysis** | Methods section: under Data analysis |
| (a) Describe all statistical methods, including those used to control for confounding | Methods section: under Data analysis |
| (b) Describe any methods used to examine subgroups and interactions | Methods section: under Data analysis |
| **Results** |  |
| Response rate reported | Results section: under Sociodemographic and academic variables of the medical students |
| Description of the participants | Results section: under Sociodemographic and academic characteristics and Table 1. |
| Outcome data | Results section and Table 1-7 |
| Main results | Results section and Table 1-7 |
|  | Results section and Table 1-7 |
| Other analyses | Results section and Table 1-7 |
| **Discussion** |  |
| Results summarized referencing study objectives | First paragraph of the Discussion section |
| Interpretation | Discussion section |
| Strengths of the study stated | Discussion section: under Strengths and limitations |
| Limitations of the study stated | Discussion section: under Strengths and limitations |
| Generalizability of results discussed | Discussion section: under Strengths and limitations |
| **Ethical Quality Indicators** |  |
| Study funding reported | Declarations |
| Research Ethics Board review reported | Declarations |
| Subject consent procedures reported | Declarations |

**Reference**

1. Turk T, Elhady MT, Rashed S, Abdelkhalek M, Nasef SA, Khallaf AM, Mohammed AT, Attia AW, Adhikari P, Amin MA *et al*: **Quality of reporting web-based and non-web-based survey studies: What authors, reviewers and consumers should consider**. *PLOS ONE* 2018, **13**(6):e0194239.

2. Sharma A, Minh Duc NT, Luu Lam Thang T, Nam NH, Ng SJ, Abbas KS, Huy NT, Marusic A, Paul CL, Kwok J *et al*: **A Consensus-Based Checklist for Reporting of Survey Studies (CROSS)**. *Journal of general internal medicine* 2021.

3. von Elm E, Altman DG, Egger M, Pocock SJ, Gotzsche PC, Vandenbroucke JP, Initiative S: **The Strengthening the Reporting of Observational Studies in Epidemiology (STROBE) statement: guidelines for reporting observational studies**. *PLoS medicine* 2007, **4**(10):e296.
